# Commentary by Gácsi et al. (2023) highlights general misconceptions within the field of dog domestication and dog–wolf comparisons

**DOI:** 10.1002/ece3.10466

**Published:** 2023-09-19

**Authors:** Christina Hansen Wheat, Linn Larsson, Patricia Berner, Hans Temrin

**Affiliations:** ^1^ Department of Zoology Stockholm University Stockholm Sweden

In their commentary, Gácsi et al. (2023) direct criticism towards our study (Hansen Wheat et al., 2022) where we used the Strange Situation Test (SST) to demonstrate that human‐directed attachment behaviour, as defined in Topál et al. (2005), is expressed not only in dogs but also in wolves. We welcome this commentary, as it gives us the opportunity to correct what we consider to be misrepresentations of our study by Gácsi et al. (2023), which also includes some more general misconceptions within the field of dog domestication and dog–wolf comparisons. In this correction, we find it necessary to further draw comparisons to the original study by Topál et al. (2005), for which our study was a replication.

The main objection put forward by Gácsi et al. (2023) is that our methodology is flawed. Specifically, the authors bring forward two points: (1) our test room was not unfamiliar to the wolves and dogs, and thus allegedly failed to induce a moderate stress response, and (2) the familiar person we used was not a dedicated care taker. Because of these two points, Gácsi et al. (2023) call the validity of our results into question and claim that we have failed to demonstrate attachment behaviours in wolves. Below we address these two points and detail why none of them compromise our findings. Before doing so, we would like to state that Gácsi et al. (2023) are very focussed on the results on our dogs, seemingly missing the point that it is the results on our wolves that are important for the conclusions of this study and those results could have stood alone. We will, however, address the criticism directed at the results on both dogs and wolves.

Gácsi et al. (2023) claim that because the test room was not unfamiliar to the wolves and dogs, we were not successful in providing the stressful environment required to adequately elicit attachment behaviours. We find this claim particularly puzzling, as we specifically added a behavioural quantification of stress (i.e. pacing) during the test to assess how our animals experienced the test situation. From these results it was clear that the wolves indeed experienced stress during the test. Therefore, if induced stress is a prerequisite for the SST it was fulfilled and empirically demonstrated in our study, so the concern brought forward by Gácsi et al. (2023) is unfounded. Again, we emphasize that it is the results for the wolves that are important for our findings. Gácsi et al. (2023) also claim that the lack of stress behaviour in dogs is an indication that the attachment system was not sufficiently activated by the test situation because the test room was familiar. First, we did see pacing (i.e. stress behaviour) in some of our dogs equal to that of wolves, as documented in the supplementary materials. However, upon overall species comparisons, the wolves were significantly more stressed than the dogs, a finding that should not be particularly surprising given the documented physiological alterations of the HPA‐axis between non‐domesticated and domesticated animals (Künzl & Sachser, 1999; Trut et al., 2004). Second, it is well‐documented that the seemingly absence of stress behaviours does not necessarily equate the absence of a physiological stress response (Koolhaas et al., 2010; Qu et al., 2018; Schjolden et al., 2005). An additional measure of physiological stress on our wolves and dogs would have resulted in a higher resolution for our stress response quantification. In hindsight, this would have been a nice addition to our study. Together, as we demonstrated that both wolves and dogs discriminated between the familiar person and the stranger, we find Gácsi et al's. (2023) claim that the test situation was not stressful enough to elicit attachment behaviours to be unfounded. We would also like to take this opportunity to point out that in the original study by Topál et al. (2005), there was no attempt to quantify stress in either wolves or dogs. Thus, the authors did not quantitatively document that any of their studied animals actually experienced the moderate level of stress required to elicit attachment behaviours. Using the same level of rigour for the Topál et al. (2005) study, as Gácsi et al. (2023) request for ours, it must then naturally follow that those results are invalid.

The other main point of critique Gácsi et al. (2023) direct at our study, is our choice of familiar person for the test. Specifically, they claim that because our wolves and dogs were hand‐raised in litters by multiple caregivers, the familiar person used in our study was not a so‐called ‘dedicated caretaker’, and a proper attachment between her and the puppies could therefore not have taken place. We find this argument as puzzling as the one put forward regarding our use of test room, as we provide empirical evidence that quantifiably stressed wolves differentiate between a familiar person and a stranger in the test. The very essence of quantifying attachment is the test subject's ability to discriminate between an attachment figure and a stranger (Ainsworth & Bell, 1970; Rehn et al., 2013; Topál et al., 1998). Additionally, our wolves expressed significantly lower levels of stress behaviour upon reunion with the familiar person, thus only strengthening the evidence that they indeed discriminated between the stranger and their caregiver. Finally, as already outlined in the methods, the familiar person in question was the caregiver who spent the absolute most time with the puppies throughout the duration of the project and had daily prolonged contact with both the wolves and dogs. In fact, we find that using the same familiar person for all our wolves and dogs adds rigour to our results through increased environmental control. Individual hand‐raising by designated caregivers in private homes, as implemented for the wolves and dogs in Topál et al. (2005), introduces a potential environmental bias that must not be ignored. Specifically, attachment style of individual owners (e.g. confident vs. non‐confident) has been demonstrated to affect the behaviours of their dogs in the SST (Siniscalchi et al., 2013). Likewise, how dogs choose to seek support from their owners in stressful situations depend on their owner's attachment style (i.e. confident, anxious or avoidant, Rehn et al., 2017). It can therefore not be ruled out that the hand‐raising regime implemented in Topál et al. (2005) affected the wolves and dogs' performance in the SST due to the attachment style of the individual hand‐raiser, thus affecting the results. This provides a strong argument for standardizing the familiar person used in the SST as we did.

We also would like to distance ourselves from the view of Gácsi et al. (2023) that it would be feasible to engage in a hand‐raising set‐up where wolves were hand‐raised individually with the aim of quantifying attachment in exclusive caregiver–wolf dyads as done in Topál et al. (2005). Such individual rearing of highly social animals without the opportunity to continuously interact with conspecifics is considered unethical according to international standards for animal keeping (World Association for Zoos and Aquariums; European Association for Zoos and Aquariums, 2014) and national legislation for ethical scientific conduct in Sweden (e.g. SJVFS, 2019:29). Specifically, it can have devastating welfare consequences for an animal to not be raised with adequate access to conspecifics during the sensitive period (Evans, 1967; Fox, 1969; Meder, 1989; Price & Wallach, 1990). We therefore find it very concerning if individual rearing of wolves is considered by present‐day researchers. In our study we adhered to well‐established and standardized methods for hand‐raising and socialization (Klinghammer & Goodman, 1987; Range & Virányi, 2011) developed over the past 40 years, which were approved by a national Ethics Committee as explicitly described in our methods section. Additionally, Gácsi et al.'s (2023) statement about whether our wolves had been successfully socialized, as they showed fear towards the stranger, is uncalled for. It is well‐documented that wolves do not generalize their socialization to strangers (Klinghammer & Goodman, 1987; Zimen, 1987) and a recent study even details how some wolves hand‐raised according to the established method referred to above could not be tested with an unfamiliar person due to excessive fear responses (Salomons et al., 2021). We also find the comment questioning the adequate socialization of our wolves by Gácsi et al. (2023) particularly peculiar since the hand‐raised wolves in Topál et al. (2005) received less socialization compared to the hand‐raised dogs. Specifically, the wolves were relocated to an animal park up to 2 months before they were tested in the SST while the dogs kept living with their caregivers (Hall et al., 2015; Virányi et al., 2008) introducing potential environmental bias. This is problematic for the interpretation of the results, as it is well‐established that different environments affect behavioural development (Bray et al., 2017; Clark & Galef Jr., 1982; Wilsson & Sundgren, 1998; Zimen, 1987) and experimental outcomes when comparing wolves and dogs (Hare et al., 2002; Udell et al., 2008). Taken together, it is therefore profoundly surprising to us that Gácsi et al. (2023) question the method by which we have socialized our wolves.

Additionally, we would like to address why our choice of dog type is not unsuitable as claimed by Gácsi et al. (2023), simultaneously covering general misconceptions within the field of dog domestication and dog‐wolf comparisons. First, we re‐emphasize that because we and others document human‐directed attachment behaviours in wolves, despite the small sample size, these findings suggests that this ability existed as standing variation in pre‐domestication wolf populations. Albeit only demonstrated in a few wolves, these significant observations are a positive confirmation that in principle can stand alone and fundamentally refute the uniqueness of this trait to dogs. However, as we believe the results are still interesting, we chose to include the comparison with dogs in our study. We find the critique of our use of Alaskan huskies stimulating as is provides a good foundation to discuss the importance of how the choice of dogs can potentially affect results and why this choice, when used for behavioural comparison with wolves to uncover the evolutionary basis of dog behaviour, needs careful consideration. Of the worlds' approximately 400 registered dog breeds, nearly all originated in Europe less than 200 years ago (i.e. modern breeds), while a minority originated ~500 years ago (i.e. ancient breeds; Lindblad‐Toh et al., 2005; Parker et al., 2017; Vonholdt et al., 2010). In our study we used a dog type made up of ancient dog breeds. By doing so we significantly reduced the risk of post‐domestication improvement confounding our behavioural expression observations (Hansen Wheat et al., 2019, 2020; Larson & Fuller, 2014). Since most dog‐wolf comparative studies use modern breeds that have undergone extensive artificial selection to fulfil highly specialized morphological and behavioural individual breed standards, behavioural studies using such breeds likely confound post‐domestication breed improvements with original domestication phenotypes. For example, a large‐scale study using more than 76,000 dogs spanning 71 breeds demonstrated how behavioural correlations related to domestication break down in modern breeds while they remain intact in ancient breeds (Hansen Wheat et al., 2019). Furthermore, rigorous genomics and behavioural genomics studies now confirm that modern breeds are poor representatives of original dogs from a behavioural and genetic perspective (Shannon et al., 2015), as behavioural diversification predates breed formation (Dutrow et al., 2022; Morrill et al., 2022). Thus, contrary to popular beliefs, including the ones expressed by Gácsi et al. (2023), the value of comparing wolves with modern dog breeds, or mixed breed dogs of unknown origin, in efforts to understand behavioural evolution during dog domestication is of limited value. This is because the use of modern breeds results in findings for which it is impossible to disentangle whether behavioural traits expressed are breed‐specific products of targeted, artificial selection post‐breed formation, unique to the domestication process, or if they were the target of domestication that existed as variation within pre‐domestication wolf populations. In sum, phylogenetically basal dog breeds or types should be preferred, as such dogs are closer representatives of original dogs untainted by the profound artificial selection pressures and contaminant massive population bottlenecks inherent in modern breed dogs.

Lastly, we want to bring forward that Gácsi et al. (2023) mention the uniqueness of the dog–human attachment bond. Here, the authors seem to assume that the lack of evidence equals proof of concept. While true that research on the dog–human bond is significantly more common than that of wolf–human bond, we must not equate the significantly larger study pool of dogs available with unique results (Hansen Wheat & Wynne, 2023). The notoriously small sample sizes of wolves available will always pose a problem in order to adequately test hypotheses about domestication under properly controlled conditions. Importantly, here proof of concept is per definition the positive confirmation that a trait exists in wolves and this existence is independent of sample size. It is well‐established that evolutionary change relies heavily on standing genetic variation (Barrett & Schluter, 2008; Larson et al., 2014) and recent behavioural genomics research supports how central dog behaviours likely were present in ancestral wolf populations (Dutrow et al., 2022). Successfully identifying standing variation for behavioural traits in present‐day wolves is likely to be a rare occurrence (Hansen Wheat & Temrin, 2020; Hansen Wheat & Wynne, 2023), but it is a crucial step in understanding how behaviour evolved in dogs and which selection dynamics were in play during early dog domestication. Therefore, that Gácsi et al. (2023) would dismiss that wolves, in spite of now four independent studies showing otherwise, have the ability to express attachment behaviours, towards humans is concerning. We also call into question if the SST is the only method by which attachment can be adequately quantified in non‐human animals as claimed by Gácsi et al. (2023). For instance, the Secure Base Test has successfully been used to demonstrate attachment bonds between humans and domestic cats (*Felis silvestris catus*, Vitale et al., 2019), which further undermines the uniqueness narrative of dogs.

In conclusion, contrary to what Gácsi et al. (2023) claim, our standardized and environmentally controlled study design with equally socialized wolves and dogs, and the use of the same familiar person for all tested animals, provides a rigorous foundation for our results and conclusions, that is consistent with the existing literature on the existence of human‐directed attachment behaviour in wolves (Hall et al., 2015; Lenkei et al., 2020; Ujfalussy et al., 2017). While we conclude that the critique of our study is unfounded, we have found it a useful forum to discuss what we consider to be general misconceptions in the field of dog domestication research.

## AUTHOR CONTRIBUTIONS

**Christina Hansen Wheat:** Conceptualization (lead); writing – original draft (lead); writing – review and editing (lead). **Linn Larsson:** Writing – review and editing (supporting). **Patricia Berner:** Writing – review and editing (supporting). **Hans Temrin:** Writing – review and editing (supporting).

## FUNDING INFORMATION

This research did not receive any specific grants from funding agencies in the public, commercial, or not‐for‐profit sectors.

## Supporting information


Supplementary Materials

## Data Availability

There is no data in the manuscript.

## References

[ece310466-bib-0001] Ainsworth, M. D. S. , & Bell, S. M. (1970). Attachment, exploration, and separation: Illustrated by the behavior of one‐year‐Olds in a strange situation. Child Development, 41, 49–67. 5490680

[ece310466-bib-0002] Barrett, R. , & Schluter, D. (2008). Adaptation from standing genetic variation. Trends in Ecology & Evolution, 23(1), 38–44. 10.1016/j.tree.2007.09.008 18006185

[ece310466-bib-0003] Bray, E. E. , Sammel, M. D. , Cheney, D. L. , Serpell, J. A. , & Seyfarth, R. M. (2017). Effects of maternal investment, temperament, and cognition on guide dog success. Proceedings of the National Academy of Sciences of the United States of America, 114(34), 9128–9133. 10.1073/pnas.1704303114 28784785 PMC5576795

[ece310466-bib-0004] Clark, M. M. , & Galef, B. G., Jr. (1982). Environmental effects on the ontogey of exploratory and escape behaviors of Mongolian gerbils. Developmental Psychobiology, 15(2), 121–129. 10.1002/dev.420150205 7095280

[ece310466-bib-0005] Dutrow, E. V. , Serpell, J. A. , & Ostrander, E. A. (2022). Domestic dog lineages reveal genetic drivers of behavioral diversification. Cell, 185(25), 4737.e18–4755.e18. 10.1016/j.cell.2022.11.003 36493753 PMC10478034

[ece310466-bib-0006] European Association for Zoos and Aquariums . (2014). Standards for the accommodation and care of animals . https://www.eaza.net/assets/Uploads/Standards‐and‐policies/Standards‐for‐the‐Accommodation‐and‐Care‐of‐Animals‐2014.pdf

[ece310466-bib-0007] Evans, C. S. (1967). Methods of rearing and social interaction in *Macaca nemestrina* . Animal Behaviour, 15(2–3), 263–266. 10.1016/0003-3472(67)90009-7 4961875

[ece310466-bib-0008] Fox, M. W. (1969). Behavioural effects of rearing dogs with cats during the “critical period of socialization”. Behaviour, 35(3/4), 273–280.

[ece310466-bib-0009] Gácsi, M. , Miklósi, Á. , & Topál, J. (2023). Comment on "Human‐directed attachment behaviour in wolves suggest standing ancestral variation for human‐dog attachment bonds" ‐ Dogs *are* unique. Ecology and Evolution. in press. 10.1002/ece3.10514 PMC10509145 37736282

[ece310466-bib-0010] Hall, N. J. , Lord, K. , Arnold, A. M. K. , Wynne, C. D. L. , & Udell, M. A. R. (2015). Assessment of attachment behaviour to human caregivers in wolf pups (*Canis lupus lupus*). Behavioural Processes, 110, 15–21. 10.1016/j.beproc.2014.11.005 25447510

[ece310466-bib-0011] Hansen Wheat, C. , Fitzpatrick, J. L. , Rogell, B. , & Temrin, H. (2019). Behavioural correlations of the domestication syndrome are decoupled in modern dog breeds. Nature Communications, 10(1), 2422. 10.1038/s41467-019-10426-3 PMC6546797 31160605

[ece310466-bib-0012] Hansen Wheat, C. , Larson, L. , Berner, P. , & Temrin, H. (2022). Human‐directed attachment behaviour in wolves suggest standing ancestral variation for human‐dog attachment bonds. Ecology and Evolution, 12, e9299. 10.1002/ece3.9299 36188523 PMC9487184

[ece310466-bib-0013] Hansen Wheat, C. , & Temrin, T. (2020). Intrinsic ball retrieving in wolf puppies suggests standing ancestral variation for human‐directed play behavior. IScience, 23(2), 100811. 10.1016/j.isci.2019.100811 31956066 PMC7033638

[ece310466-bib-0014] Hansen Wheat, C. , van der Bijl, W. , & Wheat, C. W. (2020). Morphology does not covary with predicted behavioral correlations of the domestication syndrome in dogs. Evolution Letters, 4, 189–199. 10.1002/evl3.168 32547780 PMC7293089

[ece310466-bib-0015] Hansen Wheat, C. , & Wynne, C. D. L. (2023). The unfulfilled potential of dogs in studying behavioural evolution during the Anthropocene. Preprint on EcoEvoRxiv. 10.32942/X24G7B

[ece310466-bib-0016] Hare, B. , Brown, M. , Wiliamson, C. , & Tomasello, M. (2002). The domestication of social cognition in dogs. Science, 298(5598), 1634–1636. 10.1126/science.1072702 12446914

[ece310466-bib-0017] Klinghammer, E. , & Goodman, P. A. (1987). Socialization and Management of Wolves in captivity. In H. Frank (Ed.), Man and wolf: Advances, issues, and problems in captive wolf research (pp. 31–59). Dr W Junk Publishers.

[ece310466-bib-0018] Koolhaas, J. M. , De Boer, S. F. , Coppens, C. M. , & Buwalda, B. (2010). Neuroendocrinology of coping styles: Towards understanding the biology of individual variation. Frontiers in Neuroendocrinology, 31(3), 307–321. 10.1016/j.yfrne.2010.04.001 20382177

[ece310466-bib-0019] Künzl, C. , & Sachser, N. (1999). The behavioral endocrinology of domestication: A comparison between the domestic Guinea pig (*Cavia Apereaf*. Porcellus) and its wild ancestor, the cavy (*Cavia Aperea*). Hormones and Behavior, 35, 28–37. 10.1006/hbeh.1998.1493 10049600

[ece310466-bib-0020] Larson, G. , & Fuller, D. Q. (2014). The evolution of animal domestication. Annual Review of Ecology, Evolution, and Systematics, 45(1), 115–136. 10.1146/annurev-ecolsys-110512-135813

[ece310466-bib-0021] Larson, G. , Piperno, D. R. , Allaby, R. G. , Purugganan, M. D. , Andersson, L. , Arroyo‐Kalin, M. , Barton, L. , Climer Vigueira, C. , Denham, T. , Dobney, K. , Doust, A. N. , Gepts, P. , Gilbert, M. T. , Gremillion, K. J. , Lucas, L. , Lukens, L. , Marshall, F. B. , Olsen, K. M. , Pires, J. C. , … Fuller, D. Q. (2014). Current perspectives and the future of domestication studies. Proceedings of the National Academy of Sciences of the United States of Americ, 111(17), 6139–6146. 10.1073/pnas.1323964111 PMC4035915 24757054

[ece310466-bib-0022] Lenkei, R. , Újváry, D. , Bakos, V. , & Faragó, T. (2020). Adult, intensively socialized wolves show features of attachment behaviour to their handler. Scientific Reports, 10(1), 17296. 10.1038/s41598-020-74325-0 33057050 PMC7560749

[ece310466-bib-0023] Lindblad‐Toh, K. , Wade, C. M. , Mikkelsen, T. S. , Karlsson, E. K. , Jaffe, D. B. , Kamal, M. , Clamp, M. , Chang, J. L. , Kulbokas, E. J., 3rd , Zody, M. C. , Mauceli, E. , Xie, X. , Breen, M. , Wayne, R. K. , Ostrander, E. A. , Ponting, C. P. , Galibert, F. , Smith, D. R. , DeJong, P. , … Lander, E. S. (2005). Genome sequence, comparative analysis and haplotype structure of the domestic dog. Nature, 438(7069), 803–819. 10.1038/nature04338 16341006

[ece310466-bib-0024] Meder, A. (1989). Effects of hand‐rearing on the behavioral development of infant and juvenile gorillas (Gorilla g. gorilla). Developmental Psychobiology, 22(4), 357–376. 10.1002/dev.420220404 2721818

[ece310466-bib-0025] Morrill, K. , Hekman, J. , Li, X. , McClure, J. , Logan, B. , Goodman, L. , Gao, M. , Dong, Y. , Alonso, M. , Carmichael, E. , Snyder‐Mackler, N. , Alonso, J. , Noh, H. J. , Johnson, J. , Koltookian, M. , Lieu, C. , Megquier, K. , Swofford, R. , Turner‐Maier, J. , … Karlsson, E. K. (2022). Ancestry‐inclusive dog genomics challenges popular breed stereotypes. Science, 376(6592), eabk0639. 10.1126/science.abk0639 35482869 PMC9675396

[ece310466-bib-0026] Parker, H. G. , Dreger, D. L. , Rimbault, M. , Davis, B. W. , Mullen, A. B. , Carpintero‐Ramirez, G. , & Ostrander, E. A. (2017). Genomic analyses reveal the influence of geographic origin, migration, and hybridization on modern dog breed development. Cell Reports, 19(4), 697–708. 10.1016/j.celrep.2017.03.079 28445722 PMC5492993

[ece310466-bib-0027] Price, E. O. , & Wallach, S. J. R. (1990). Physical isolation of hand‐reared Hereford bulls increases their aggressiveness toward humans. Applied Animal Behaviour Science, 27(3), 263–267. 10.1016/0168-1591(90)90061-H

[ece310466-bib-0028] Qu, J. , Fletcher, Q. E. , Réale, D. , Li, W. , & Zhang, Y. (2018). Independence between coping style and stress reactivity in plateau Pika. Physiology & Behavior, 197, 1–8. 10.1016/j.physbeh.2018.09.007 30236525

[ece310466-bib-0029] Range, F. , & Virányi, Z. (2011). Development of gaze following abilities in wolves (*Canis lupus*). PLoS One, 6(2), e16888. 10.1371/journal.pone.0016888.s002 21373192 PMC3044139

[ece310466-bib-0030] Rehn, T. , Beetz, A. , & Keeling, L. J. (2017). Links between an owner's adult attachment style and the support‐seeking behavior of their dog. Frontiers in Psychology, 8, 237–300. 10.1080/00221325.2014.885879 29250009 PMC5715226

[ece310466-bib-0031] Rehn, T. , McGowan, R. T. S. , & Keeling, L. J. (2013). Evaluating the strange situation procedure (SSP) to assess the bond between dogs and humans. PLoS One, 8(2), e56938. 23437277 10.1371/journal.pone.0056938 PMC3577677

[ece310466-bib-0032] Salomons, H. , Smith, K. C. M. , Callahan‐Beckel, M. , Callahan, M. , Levy, K. , Kennedy, B. S. , Bray, E. E. , Gnanadesikan, G. E. , Horschler, D. J. , Gruen, M. , Tan, J. , White, P. , von Holdt, B. M. , MacLean, E. L. , & Hare, B. (2021). Cooperative communication with humans evolved to emerge early in domestic dogs. Current Biology, 31(14), 3137.e11–3144.e11. 10.1016/j.cub.2021.06.051 34256018 PMC8610089

[ece310466-bib-0033] Schjolden, J. , Backstrom, T. , Pulman, K. , Pottinger, T. , & Winberg, S. (2005). Divergence in behavioural responses to stress in two strains of rainbow trout (*Oncorhynchus mykiss*) with contrasting stress responsiveness. Hormones and Behavior, 48(5), 537–544. 10.1016/j.yhbeh.2005.04.008 15907328

[ece310466-bib-0034] Shannon, L. M. , Boyko, R. H. , Castelhano, M. , Corey, E. , Hayward, J. J. , McLean, C. , White, M. E. , Abi Said, M. , Anita, B. A. , Bondjengo, N. I. , Calero, J. , Galov, A. , Hedimbi, M. , Imam, B. , Khalap, R. , Lally, D. , Masta, A. , Oliveira, K. C. , Pérez, L. , … Boyko, A. R. (2015). Genetic structure in village dogs reveals a central Asian domestication origin. Proceedings of the National Academy of Sciences of the United States of America, 112(44), 13639–13644. 10.1073/pnas.0909559107 26483491 PMC4640804

[ece310466-bib-0035] Siniscalchi, M. , Stipo, C. , & Quaranta, A. (2013). “Like owner, like dog”: Correlation between the Owner's attachment profile and the owner‐dog bond. PLoS One, 8(10), e78455. 10.1371/journal.pone.0078455 24205235 PMC3813613

[ece310466-bib-0036] SJVFS . (2019). 29: Statens Jordbruksverks Föreskrifter Om Djurhållning i Djurparker m.m. | Lagen.Nu. https://lagen.nu/sjvfs/2019:29

[ece310466-bib-0037] Topál, J. , Gácsi, M. , Miklósi, Á. , Virányi, Z. , Kubinyi, E. , & Csányi, V. (2005). Attachment to humans: A comparative study on hand‐reared wolves and differently socialized dog puppies. Animal Behaviour, 70(6), 1367–1375. 10.1016/j.anbehav.2005.03.025

[ece310466-bib-0038] Topal, J. , Miklósi, Á. , Csanyi, V. , & Doká, A. (1998). Attachment behavior in dogs (*Canis familiaris*): A new application of Ainsworth's (1969) strange situation test. Journal of Comparative Psychology, 112(3), 219–229. 9770312 10.1037/0735-7036.112.3.219

[ece310466-bib-0039] Trut, L. N. , Plyusnina, I. Z. , & Oskina, I. N. (2004). An experiment on Fox domestication and debatable issues of evolution of the dog. Russian Journal of Genetics, 40(6), 644–655. 10.1023/B:RUGE.0000033312.92773.c1 15341270

[ece310466-bib-0040] Udell, M. A. R. , Dorey, N. R. , & Wynne, C. D. L. (2008). Wolves outperform dogs in following human social cues. Animal Behaviour, 76(6), 1767–1773. 10.1016/j.anbehav.2008.07.028

[ece310466-bib-0041] Ujfalussy, D. J. , Kurys, A. , Kubinyi, E. , Gácsi, M. , & Virányi, Z. (2017). Differences in greeting behaviour towards humans with varying levels of familiarity in hand‐reared wolves (*Canis lupus*). Royal Society Open Science, 4(6), 160956. 10.1002/ajp.20535 28680658 PMC5493900

[ece310466-bib-0042] Virányi, Z. , Gácsi, M. , Kubinyi, E. , Topál, J. , Belényi, B. , Ujfalussy, D. , & Miklósi, Á. (2008). Comprehension of human pointing gestures in Young human‐reared wolves (*Canis lupus*) and dogs (*Canis familiaris*). Animal Cognition, 11(3), 373–387. 10.1007/s10071-007-0127-y 18183437

[ece310466-bib-0043] Vitale, K. R. , Behnke, A. C. , & Udell, M. A. R. (2019). Attachment bonds between domestic cats and humans. Current Biology, 29(18), R864–R865. 10.1016/j.cub.2019.08.036 31550468

[ece310466-bib-0044] Vonholdt, B. M. , Pollinger, J. P. , Lohmueller, K. E. , Han, E. , Parker, H. G. , Quignon, P. , Degenhardt, J. D. , Boyko, A. R. , Earl, D. A. , Auton, A. , Reynolds, A. , Bryc, K. , Brisbin, A. , Knowles, J. C. , Mosher, D. S. , Spady, T. C. , Elkahloun, A. , Geffen, E. , Pilot, M. , … Wayne, R. K. (2010). Genome‐wide SNP and haplotype analyses reveal a rich history underlying dog domestication. Nature, 464(7290), 898–902. 10.1038/nature08837 20237475 PMC3494089

[ece310466-bib-0045] Wilsson, E. , & Sundgren, P. E. (1998). Behaviour test for eight‐week old puppies—Heritabilities of tested behaviour traits and its correspondence to later behaviour. Applied Animal Behaviour Science, 58, 151–162.

[ece310466-bib-0046] Zimen, E. (1987). Ontogeny of approach and flight behavior towards humans in wolves, poodles and wolf‐poodle hybrids. In H. Frank (Ed.), Man and wolf: Advances, issues, and problems in captive wolf research (pp. 275–292). Dr W. Junk Publishers.

